# Molecular-based detection of potentially pathogenic bacteria in membrane bioreactor (MBR) systems treating municipal wastewater: a case study

**DOI:** 10.1007/s11356-016-8211-y

**Published:** 2016-12-24

**Authors:** Moustapha Harb, Pei-Ying Hong

**Affiliations:** Water Desalination and Reuse Center, Environmental Science and Engineering, King Abdullah University of Science and Technology (KAUST), 4700 King Abdullah Boulevard, Thuwal, 23955-6900 Saudi Arabia

**Keywords:** Pathogens, Removal rates, Aerobic, Anaerobic, Reuse, Bioreactor, Wastewater

## Abstract

Although membrane bioreactor (MBR) systems provide better removal of pathogens compared to conventional activated sludge processes, they do not achieve total log removal. The present study examines two MBR systems treating municipal wastewater, one a full-scale MBR plant and the other a lab-scale anaerobic MBR. Both of these systems were operated using microfiltration (MF) polymeric membranes. High-throughput sequencing and digital PCR quantification were utilized to monitor the log removal values (LRVs) of associated pathogenic species and their abundance in the MBR effluents. Results showed that specific removal rates vary widely regardless of the system employed. Each of the two MBR effluents’ microbial communities contained genera associated with opportunistic pathogens (e.g., *Pseudomonas*, *Acinetobacter*) with a wide range of log reduction values (< 2 to >5.5). Digital PCR further confirmed that these bacterial groups included pathogenic species, in several instances at LRVs different than those for their respective genera. These results were used to evaluate the potential risks associated both with the reuse of the MBR effluents for irrigation purposes and with land application of the activated sludge from the full-scale MBR system.

**Electronic supplementary material:**

The online version of this article (doi:10.1007/s11356-016-8211-y) contains supplementary material, which is available to authorized users.

## Introduction

The issue of pathogen presence in treated wastewater effluents has gained attention recently due to an increased interest in reuse applications (Li et al. 2013; Zanetti et al. 2010). Previous studies have highlighted the advantages of aerobic membrane bioreactor (MBR) systems for the removal of microbial indicator bacteria (i.e. *Escherichia coli*, total coliforms, fecal coliforms) from effluent discharges (Francy et al. 2012; Hai et al. 2014; Ottoson et al. 2006). Despite the high quality and low particulate effluents produced by MBR systems, it has been observed that 100% rejection of bacteria is not achievable by MBRs when operated with microfiltration (MF) membranes and that log removal rates (LRVs) vary based on the microbial indicator detected (Jong et al. 2010; Trinh et al. 2012; van den Akker et al. 2014). This variability in microbial removal rates (<10^4^ to >10^6^ removal) poses an obstacle for reuse purposes, as it means that chlorine disinfection remains necessary for post-MBR effluents. Chlorination substantially reduces microbial risk, but toxic and carcinogenic disinfection by-products formed by chlorination can have a deleterious effect on effluents being applied for reuse (Krasner et al. 2009; Richardson et al. 2007).

An additional issue associated with aerobic MBRs, and activated sludge processes in general, is that of sludge production and disposal. Despite land application of sewage sludge being widely used throughout the world, pathogen-associated health effects of this practice are still of significant concern (Lewis and Gattie 2002; Lowman et al. 2013). This is, in part, due to inadequate treatment of sewage sludge before land application or disposal, especially in developing and industrialized countries (Pérez-Elvira et al. 2006). For example, a recent study assessing wastewater treatment practices in China found that the vast majority of sludge treatment processes consisted of only sludge thickening and mechanical dewatering (Jin et al. 2014).

Given the limitations of aerobic MBRs, anaerobic MBRs (AnMBRs) have been viewed as a potential alternative municipal wastewater treatment technology due to their low sludge production rates, low energy use, and nutrient-rich effluents (Smith et al. 2012). However, due to the lack of full-scale systems in operation, research addressing the microbial removal efficiencies of AnMBRs has been limited (Ellouze et al. 2009; Wong et al. 2009). Despite the inherently different effluent water composition (i.e., nutrient content) produced from AnMBRs compared to aerobic MBR effluents, there have not yet been any studies examining how these differences would impact the bacterial communities released into the environment. More specifically, there is a need to understand if and how the pathogenic bacteria present in wastewater influents would persist through AnMBR systems into their effluents.

A wide range of pathogenic bacteria are known to be present in municipal wastewater (Cai et al. 2014; Cai and Zhang 2013; Ye and Zhang 2011). Given that significant variability has been observed in the removal rates of indicator bacteria by MBRs in previous studies (Zanetti et al. 2010), a systematic assessment based on comprehensive molecular-based detection is therefore needed to determine the removal efficacies of aerobic and anaerobic MBRs. In particular, the use of high-detection sensitivity methods such as high-throughput sequencing and digital PCR would be useful in addressing the removal of pathogens by MBRs (Bian et al. 2015; Cai and Zhang 2013).

The purpose of the present study is to employ high-throughput and digital PCR approaches to examine the specific presence and removal of potentially pathogenic bacteria in municipal wastewater by a full-scale aerobic MBR plant and a lab-scale anaerobic MBR system. It was further intended to apply results obtained from the molecular-based detection of specific pathogenic bacteria to evaluate the risks associated with both the reuse of MBR effluents and the disposal/application of the aerobic MBR and activated sludge using quantitative microbial risk assessment (QMRA).

## Materials and methods

### Full-scale aerobic MBR system description and sampling protocol

**Fig. 1:**
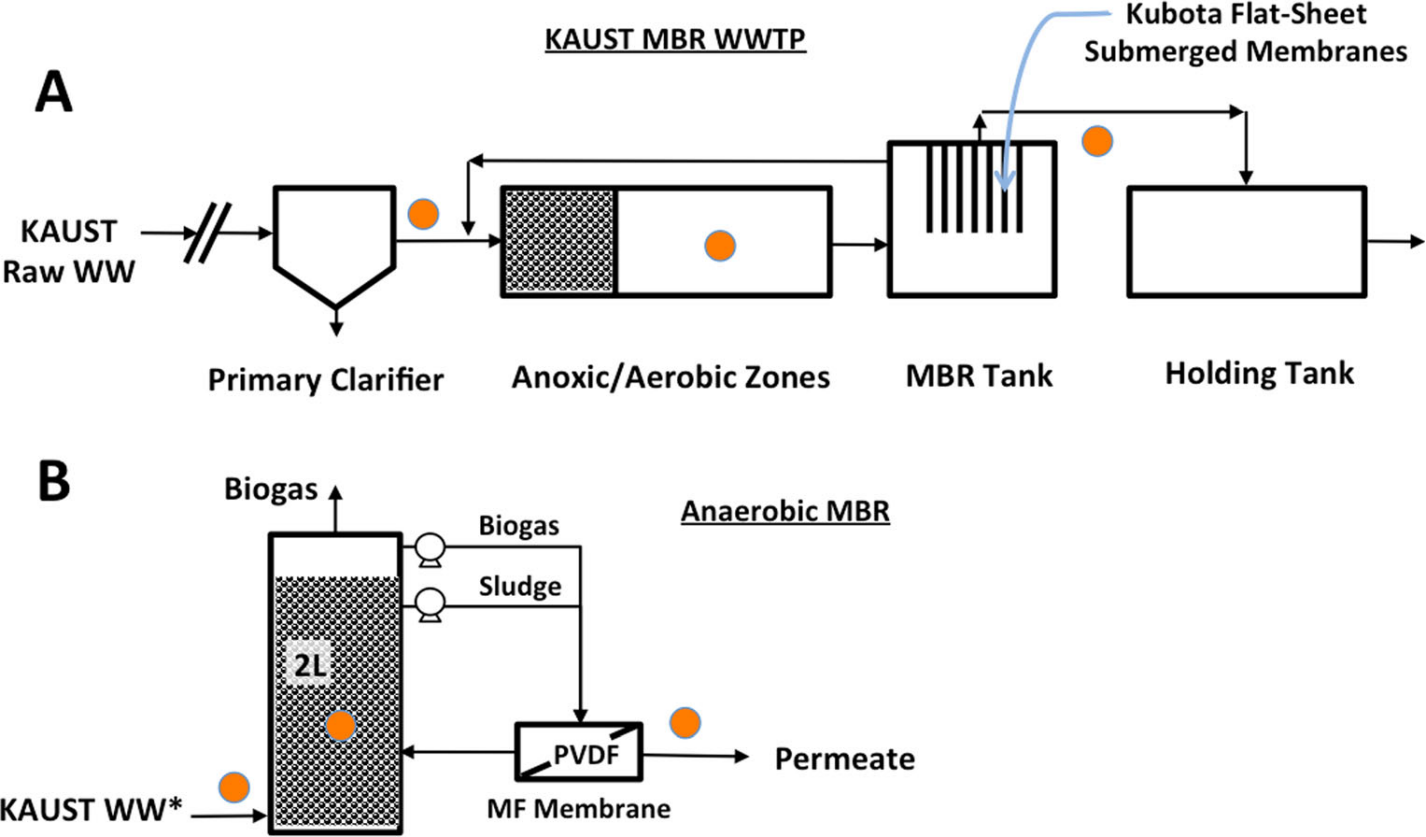
Schematic diagrams of both MBR systems sampled in this study. **a** Schematic of full-scale AeMBR WWTP. Sampling points are indicated by *orange dots* and include (1) post-clarification influent, (2) aerobic activated sludge, and (3) MBR effluent. **b** Schematic of lab-scale AnMBR. Sampling points are indicated by *orange dots* and include (1) post-clarification influent, (2) anaerobic sludge, and (3) AnMBR effluent

The aerobic MBR evaluated in this study was a full-scale wastewater treatment plant receiving 6700 m^3^/day of raw wastewater. The full-scale aerobic MBR (AeMBR) system consisted of the following process units: (i) primary clarifier, (ii) anoxic and aerobic activated sludge tanks, (iii) submerged membrane tank, and (iv) holding tank (Fig. 1a). Membranes employed were flat-sheet 0.4-μm nominal pore-sized MF membrane cartridges by Kubota Membrane (Kubota Corporation, Osaka, Japan). A detailed description of the system operating conditions is provided in Appendix S1. Sampling was conducted between March 2015 and January 2016. Samples were collected from the influent, activated sludge, and MBR effluent as indicated in Fig. 1a. Influent samples were prepared by centrifuging 30 to 100 mL of influent at 9400×*g* for 10 min to obtain a biomass pellet while effluent samples were prepared by filtering 2 L through 0.4-μm Whatman Nuclepore™ track-etched polycarbonate membrane filters (GE Healthcare Life Sciences, Little Chalfont, UK) to retain the biomass. The filters with the retained biomass were subsequently used for DNA extraction. Finally, activated sludge samples were obtained by mixing 0.2 mL of sludge with 0.8 mL of 1× PBS solution and centrifuging at 9400×*g* for 10 min.

### Lab-scale anaerobic MBR system description and sampling protocol

The anaerobic MBR (AnMBR) used in this study was a mesophilic up-flow attached-growth (UA) 2-L anaerobic reactor as described previously (Harb et al. 2015). The reactor was connected in external cross-flow configuration to a 0.3-μm nominal pore-sized polyvinylidene difluoride (PVDF) MF membrane (Fig. 1b). The system was fed with the same municipal wastewater being treated by the full-scale MBR plant. A detailed description of the reactor operational conditions is provided in Appendix S1. Sampling was conducted over a 9-month period from April 2015 to January of 2016. The AnMBR effluent was sampled by filtering 0.5 L through 0.4-μm Whatman Nuclepore™ track-etched polycarbonate membrane filters (GE Healthcare Life Sciences, Little Chalfont, UK) to retain the biomass. The filters with the retained biomass were subsequently used for DNA extraction. The AnMBR was also sampled for suspended and attached biomass using protocols described previously (Harb et al. 2015).

### Water quality and biogas measurements

Water quality was monitored for both the AeMBR and AnMBR influents and effluents by measuring chemical oxygen demand (COD), ammonia, nitrate, and nitrite content. COD of influent and effluent samples was measured using either LCK 314 (15–150 mg/L) or LCK 514 COD (100–2000 mg/L) cuvette test vials depending on the concentration to be measured. NH_4_^+^-N, NO_2_^−^-N, and NO_3_^−^-N concentrations were measured using Test ‘N Tube high range ammonia kit, TNTplus 839, and TNTplus 835, respectively. All measurements were conducted based on protocols specified by the manufacturer (Hach Lange, Manchester, UK). Biogas produced from the AnMBR was captured continuously in gas bags from the headspace of the reactor and measured for volume, CH_4_, O_2_, N_2_, and H_2_ as described previously (Harb et al. 2015).

### DNA extraction and 16S rRNA gene-based next-generation sequencing

Biomass used for DNA extraction was obtained from 0.2 mL of sludge, 50 mL of influent, 0.5 L of AnMBR effluent, and 2 L of AeMBR effluent for each sample. The varying volumes used for samples obtained at different stages of the wastewater treatment process is because of the need to obtain an approximate biomass weight that is similar across samples, given that an earlier study has shown that differences in initial biomass weight prior to DNA extraction can result in differences in microbial community analysis (Molbak et al. 2006). Genomic DNA was extracted using the UltraClean Soil DNA Isolation Kit (MO BIO Laboratories, Carlsbad, USA) with slight modifications to the manufacturer’s protocol (Hong et al. 2011). Briefly, cells were lysed by adding 10 μl of 100 mg/mL lysozyme and 10 μl of 1 mg/mL achromopeptidase to the extraction buffer and incubated at 37 °C for 1 h. Both lysozyme and achromopeptidase break down the β-1,4-glycosidic bonds in the peptidoglycan of bacterial cell walls, thus enhancing cell lysis. After incubation, samples were further processed by mechanical lysis using bead-based vortexing. Samples were then pelleted at 9400×*g* for 1 min to remove biomass particles from the extracted DNA in solution. Supernatant was loaded onto spin filters by centrifugation at 9400×*g* for 1 min and washed with an ethanol-based solution. The DNA was then eluted from the spin filters with 40 μl of molecular-grade water. Illumina MiSeq amplicon sequencing was performed to provide information on the total microbial community. Details of the primers, PCR protocol, and quality control are presented in Appendix S2. Purified amplicons were submitted to the KAUST Genomics Core Lab for unidirectional sequencing on an Illumina MiSeq platform. Raw sequence reads were filtered to remove those determined to be chimeras and those with lengths of <300 nt. Filtered sequence reads were analyzed using a Ribosomal Database Project (RDP) classifier and an operational taxonomic unit (OTU)-based protocol as described previously (Harb et al. 2015). Relative abundances based on these sequence reads were adjusted by the RDP classifier for 16S ribosomal RNA (rRNA) gene copy numbers per cell based on data from genome sequences obtained from the Ribosomal RNA Database (rrnDB) (Stoddard et al. 2014). Subsequently, relative abundance of each genus was estimated by normalizing the adjusted read numbers assigned to each genus against the total reads obtained for that sample. All high-throughput sequencing files were deposited in the European Nucleotide Archive (ENA) under study accession number PRJEB14612.

### Species-targeted digital PCR

Digital PCR (dPCR) was performed to determine the relative abundances of species associated with opportunistic pathogens in influent, effluent, and sludge samples. dPCR was performed using primers targeting *Acinetobacter baumannii* (McConnell et al. 2012) (*ompA*), *Klebsiella pneumoniae* (Lee et al. 2006) (*phoE*), and *Pseudomonas aeruginosa* (Lee et al. 2006) (*regA*). Relative gene abundances were normalized per liter of sample. *rpoB* gene copy numbers were also quantified to estimate total bacterial cell counts on the basis of single-copy gene homogeneity in all bacterial species (Dahllöf et al. 2000). dPCR was performed using the Clarity digital PCR System with a 32-tube reader (JN Medsys, Singapore) based on the manufacturer’s instructions. A description of the primers, dPCR protocol, detection sensitivity, and thermal cycling programs used is presented in Appendix S3. Primer sequences and their associated target species are shown in Table S1.

### Quantitative microbial risk assessment

To further evaluate the potential microbial risks arising from reuse of the effluents of both AeMBR and AnMBR, QMRA was performed for the three pathogenic species previously detected by dPCR. Additionally, the disposal of dewatered activated sludge was evaluated by QMRA for microbial risk of human exposure for *A. baumannii* and *K. pneumoniae* due to their detection in the AeMBR activated sludge. Bacterial cell counts for each pathogenic species were estimated based on *ompA*, *phoE*, *and regA* all being single-copy genes (Fitch et al. 1993; Hedstrom et al. 1986; Martiny et al. 2006). Probability of transmission of the bacteria was calculated based on an assumed value of 2.0 × 10^−6^ (Gerba and Choi 2006). QMRA was performed based on the main induction route for agricultural workers being dermal exposure to liquid particulates during irrigation events and induction to individuals being through dermal contact with sludge and accidental ingestion of particulates during land applying and/or land filling dewatered activated sludge. The individuals potentially exposed to the sludge include workers involved in land application/disposal and other persons possibly entering disposal sites (Harder et al. 2014). Exposure risks associated with aerosol ingestion during irrigation were not incorporated into this assessment due to the minimum enteric cell concentration in solution required for aerosolized detection (>10^6^/L) being above those measured in both effluents and due to the high variability of the route’s associated exposure factors (Blumenthal et al. 2000).

Exposure assessment parameters were obtained from the USEPA exposure factor handbook (USEPA 2011). The *k* constants used for opportunistic pathogens were 2.76 × 10^−7^ for *A. baumannii* (López-Rojas et al. 2011), 1.05 × 10^−4^ for *P. aeruginosa* (Hazlett et al. 1978), and 1.62 × 10^−6^ for *K. pneumoniae* (Domenico et al. 1982) as determined by their LD_50_ dose based on an exponential model.

Point risk estimates were calculated using the following equation:

$$ \mathrm{Point}\ \mathrm{risk}=1-{\mathrm{e}}^{\left(-\mathrm{k}*\mathrm{exposed}\ \mathrm{dose}\right)} $$

Annual risk estimates were further calculated using the following:

$$ \mathrm{Annual}\ \mathrm{risk}=1-{\left(1-\mathrm{point}\ \mathrm{risk}\right)}^{\mathrm{number}\ \mathrm{of}\ \mathrm{exposure}\ \mathrm{days}\ \mathrm{per}\ \mathrm{year}} $$

Annual risk was evaluated based on an acceptable microbial risk of 1 × 10^−4^ (Smeets et al. 2009). QMRA description and calculations for exposure dosages, point risk, and annual risk values are provided in detail in Appendix S4.

## Results

### AeMBR and AnMBR water quality measurements and performance

COD removal for the AeMBR system was greater than 93% for all samples (Table S3). NH_4_^+^-N was detected in influent at an average concentration of 12.0 ± 2.8 mg/L and was undetected in AeMBR effluent samples. Conversely, nitrate but not nitrite was detected consistently in effluent samples at an average concentration of 15.2 ± 2.7 mg/L NO_3_-N, implying full nitrification by the system.

The AnMBR showed COD removal rates of 95–98% throughout operation (Table S4). Ammonia was detected in influent wastewater at an average concentration of 252 ± 4 mg/L NH_4_^+^-N while neither nitrite nor nitrate was detected. AnMBR effluent contained an average concentration of 242 ± 5 mg/L NH_4_^+^-N and no nitrate or nitrite, showing no nitrogen conversion by the AnMBR. The biogas produced by the AnMBR contained 72–78% methane, resulting in an average methane production of 241 ± 12 mL CH_4_/g COD.

### Estimation of total bacteria by *rpoB* gene quantification

**Fig. 2:**
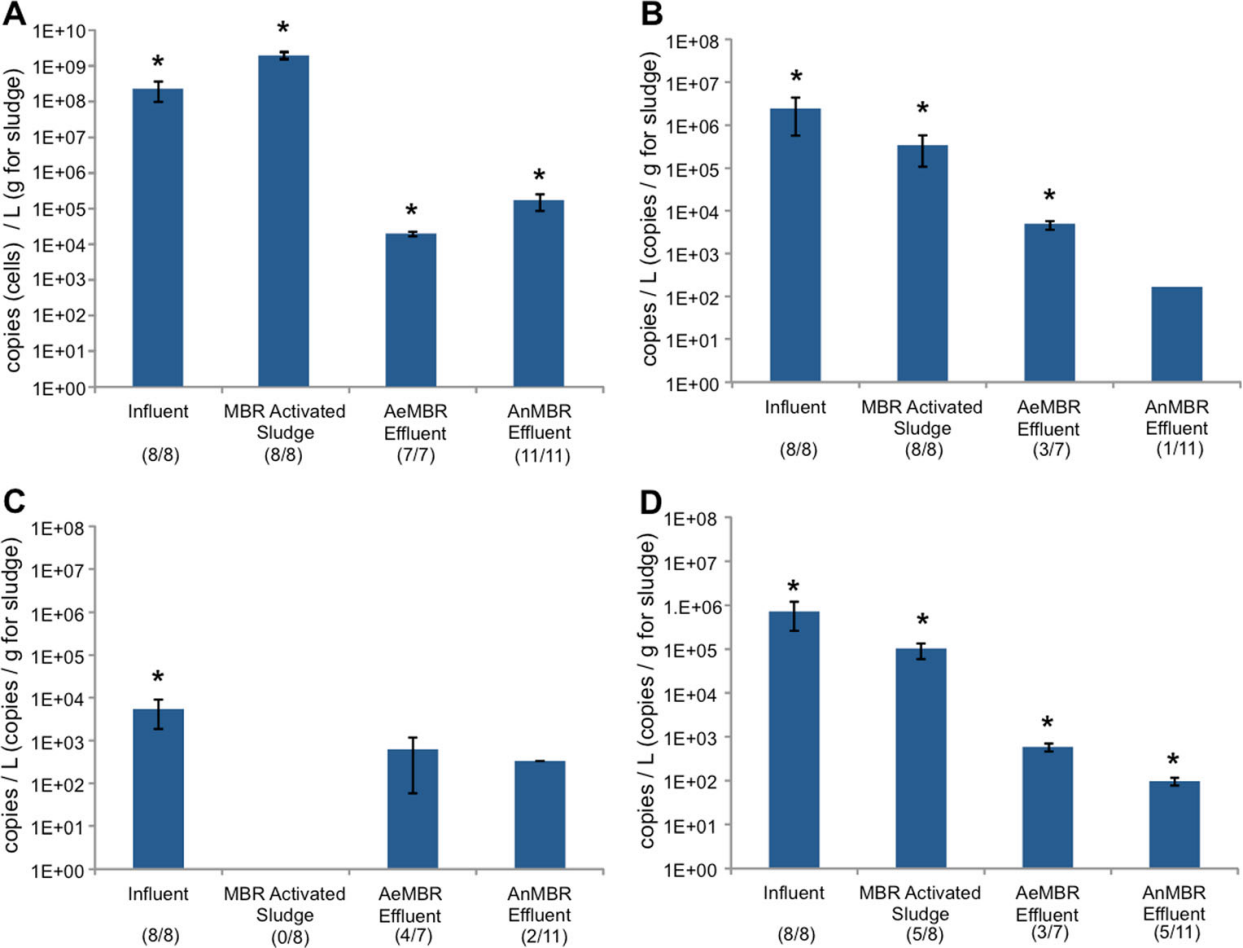
Gene abundances associated with **a** total bacteria (*rpoB*), **b** *Acinetobacter baumannii* (*ompA*), **c** *Pseudomonas aeruginosa* (*regA*), and **d** *Klebsiella pneumoniae* (*phoE*) expressed per liter of sample for wastewater influent, AeMBR effluent, and AnMBR effluent. Gene abundances of activated sludge were expressed per gram due to dewatered sludge disposal being the main source of microbial risk. Numbers of samples for which each gene was detected out of total samples are shown in *parentheses* below each *column*. Detection limits were 6 × 10^2^, 1.5 × 10^1^, 6 × 10^1^ copies per liter for influent, AeMBR effluent, and AnMBR effluent, respectively. The detection limit for AeMBR sludge was 1.6 × 10^2^ copies per gram. *Asterisks* indicate that sample groups are significantly different from all other groups of the same gene type (unpaired *t* test, *P* ≤ 0.05)

Copy numbers of the *rpoB* gene were quantified by dPCR to estimate total bacterial cell counts. Influent municipal wastewater contained an average total bacterial cell count of 2.3 × 10^8^ ± 1.2 × 10^8^ cells/L (Fig. 2a) while AeMBR and AnMBR effluent total bacteria were estimated at 1.9 × 10^4^ ± 2.7 × 10^3^ and 1.8 × 10^5^ ± 8.2 × 10^4^ cells/L, respectively. AeMBR activated sludge contained 2.6 × 10^10^ ± 3.5 × 10^9^ cells/L, which corresponded with 1.6 × 10^9^ ± 2.2 × 10^8^ cells/g (Fig. 2a). Based on these values, the AeMBR approximate LRV for total bacterial cells was 4.1 while for the AnMBR, the LRV was 3.1.

### Microbial communities of municipal wastewater influent

Relative abundances of 16S rRNA-based microbial classifications and *rpoB* gene-based total bacterial quantifications were used to estimate the levels of pathogen-associated genera in the municipal wastewater influent. The results of this analysis showed that 13 different pathogen-associated genera were identified in one or more of the wastewater influent samples. The most consistently detected genera in the influent of the MBRs included *Acinetobacter*, *Aeromonas*, *Arcobacter*, *Dialister*, *Escherichia*, *Pseudomonas*, *Stenotrophomonas*, and *Streptococcus* with at least seven of nine samples showing positive detection (Table 1).

**Table 1:** Estimated average number of cells per liter of genera associated with opportunistic pathogens

Genera	Influent Avg. (*n* = 9)	AeMBR Eff. Avg. (*n* = 8)	AnMBR Eff. Avg. (*n* = 11)	AeMBR LRV	AnMBR LRV
*Mycobacterium*	ND^	1.9 × 10^1^ (5/8)	ND	2.8	–
*Treponema*	3.3 × 10^4^ (5/9)	ND^	ND	–	–
*Arcobacter*	1.0 × 10^7^ (9/9)	2.7 × 10^1^ (7/8)	1.2 × 10^4^ (11/11)	5.6	2.9
*Neisseria*	3.4 × 10^4^ (3/9)	ND^	ND	–	–
*Acinetobacter* ^a^	1.4 × 10^7^ (9/9)	1.1 × 10^2^ (7/8)	4.7 × 10^4^ (11/11)	5.1	2.5
*Pseudomonas* ^a^	2.4 × 10^5^ (7/9)	7.7 × 10^1^ (8/8)	8.1 × 10^2^ (8/11)	3.5	2.5
*Legionella*	1.0 × 10^4^ (3/9)	2.0 × 10^1^ (7/8)	ND	2.7	–
Unclassified *Enterobacteriaceae* ^a^	1.3 × 10^6^ (9/9)	4.4 × 10^1^ (6/8)	1.8 × 10^3^ (7/11)	4.5	2.9
*Escherichia*	9.8 × 10^4^ (8/9)	ND^	ND	–	–
*Stenotrophomonas*	1.6 × 10^5^ (8/9)	2.2 × 10^1^ (6/8)	3.0 × 10^3^ (10/11)	3.9	1.7
*Aeromonas*	1.6 × 10^6^ (9/9)	8.3 × 10^0^ (5/8)	2.3 × 10^2^ (4/11)	5.3	3.9
*Streptococcus*	1.0 × 10^6^ (9/9)	8.5 × 10^0^ (4/8)	ND	5.1	–
*Enterococcus*	ND^	ND^	ND	–	–
*Dialister*	3.9 × 10^5^ (9/9)	ND^	ND	–	–

These absolute values were calculated by multiplying the copy number-adjusted 16S rRNA gene relative abundances by the total bacterial cell counts as determined by *rpoB* gene copy numbers, assuming one gene copy per bacterial cell. Log reduction values (LRVs) are shown for both the full-scale AeMBR and the lab-scale AnMBR. The numbers of samples showing positive detection are shown in parentheses

*ND* genus was not detected in any samples, *ND^* genus was detected in two or fewer samples of that type, *−*total removal

^a^Bacterial groups that were selected as targets for further investigation at the species level by digital PCR

### Microbial communities of effluents of AeMBR and AnMBR systems

**Fig. 3:**
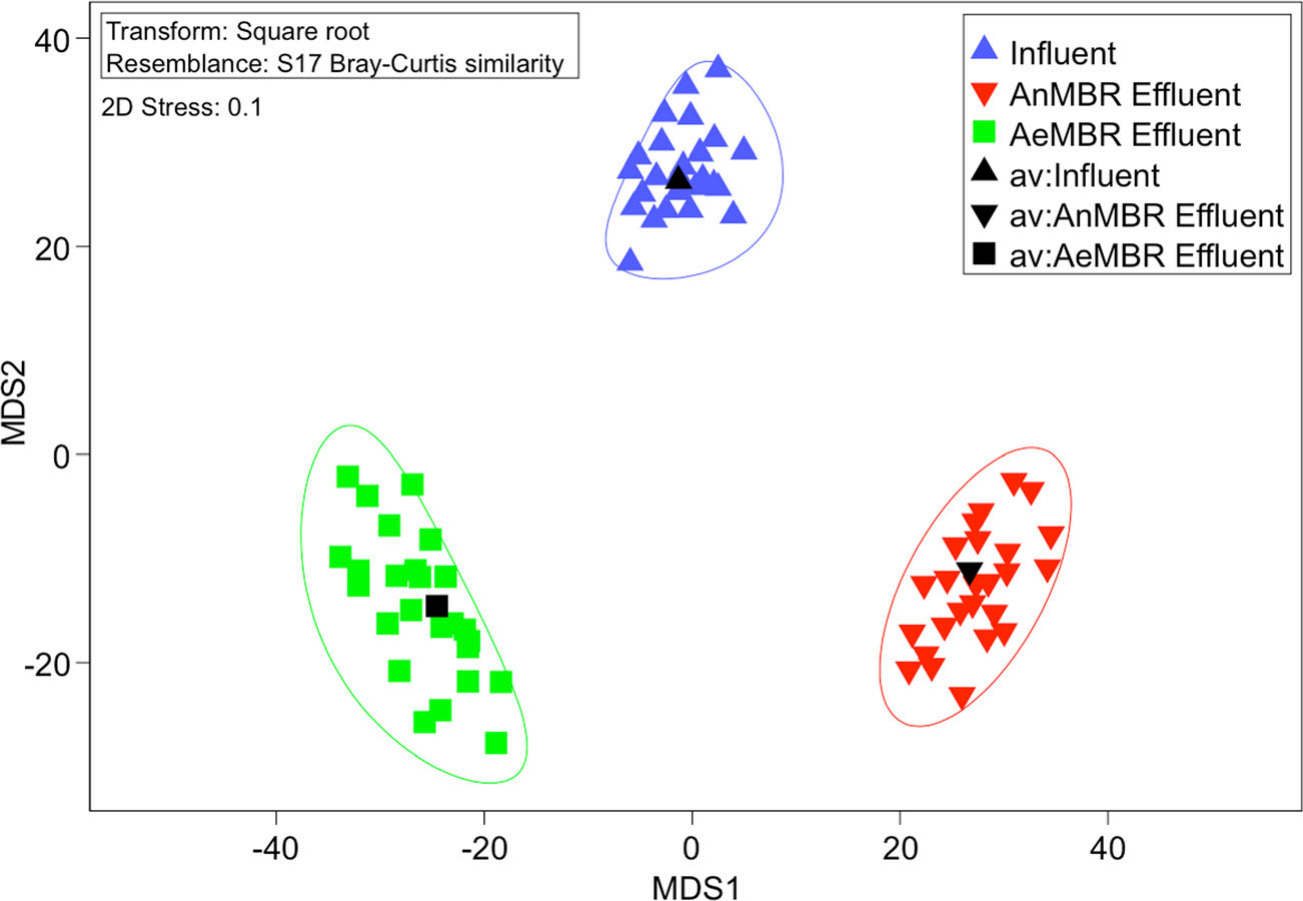
Microbial community metric multidimensional scaling plot (mMDS) for the **a** influent wastewater used for both systems, **b** full-scale AeMBR effluent, and **c** AnMBR effluent. *Black-colored symbols* represent the centroid of all samples of one type

Similarities of the microbial communities of influent samples and effluents from the full-scale AeMBR and lab-scale AnMBR reactors were calculated using Bray-Curtis similarities and represented in an mMDS plot (Fig. 3). Clustering of samples showed that effluents of both MBR systems were significantly different from the influent samples as well as from each other.

All of the genera identified in the municipal wastewater were also detected in the effluent of the full-scale AeMBR at least once (Table 1). Estimated LRVs based on samples containing the associated genera varied from 2.7 to 5.6. Genera with the highest estimated removal rates were *Acinetobacter*, *Arcobacter*, *Aeromonas*, and *Streptococcus*, all of which showed LRVs of above 5. Conversely, *Mycobacterium* and *Legionella* showed the lowest reduction rates, with LRVs of below 3.

Of the 13 pathogen-associated groups detected in the influent, 5 genera were observed in the effluent of the lab-scale AnMBR system while 8 were undetected (Table 1). Four of these 5 genera were seen consistently in the effluent with at least 8 of 11 samples showing positive detection. The detected genera included *Acinetobacter*, *Aeromonas*, *Arcobacter*, *Pseudomonas*, and *Stenotrophomonas*. Estimated LRVs for these groups were 2.5, 3.9, 2.9, 2.5, and 1.7, respectively.

### Detection of pathogenic species in MBR systems using dPCR

Given that the majority of the species associated with potentially pathogenic genera are likely nonpathogenic, further investigation of specific pathogenic species was conducted. Samples were quantified for *A. baumannii* and *P. aeruginosa*, both of which are pathogenic species for which their associated genera were found in both MBR effluents (Table 1). Due to consistent detection of unclassified *Enterobacteriaceae* in effluent samples, *K. pneumoniae* was also targeted as a representative pathogenic species within *Enterobacteriaceae*. These specific bacterial species were targeted by dPCR to determine their relative abundances per liter of the wastewater influent and full-scale AeMBR and lab-scale AnMBR effluents. The AeMBR sludge was also targeted due to the potential risk associated with disposal of the activated sludge (>600 kg produced daily at the local wastewater treatment facility) while AnMBR sludge was not tested as anaerobic systems generally require little to no sludge wastage (SRT >350 days). Pathogenic species abundances were expressed per gram of activated sludge due to the microbial risks arising from sludge disposal occurring after dewatering. Based on the determined dPCR detection limit of 0.8 copies of gene target per microliter of stock DNA, sample detection limits were 6 × 10^2^, 1.5 × 10^1^, and 6 × 10^1^ copies per liter for influent, AeMBR effluent, and AnMBR effluent, respectively, after accounting for the extracted volumes of each sample type. The detection limit for AeMBR sludge was 1.6 × 10^2^ copies per gram.

Primers targeting the *ompA* gene revealed that all wastewater influent (*n* = 8) and AeMBR activated sludge (*n* = 8) samples showed positive detection for *A. baumannii* at 2.5 × 10^6^ ± 1.9 × 10^6^ copies/L and 3.6 × 10^5^ ± 2.5 × 10^5^ copies/g, respectively (Fig. 2b). The average for the AeMBR effluent samples that showed detection was 5.0 × 10^3^ ± 1.2 × 10^3^ copies/L (three of seven). In the case of the AnMBR effluent, only 1 of 11 samples indicated the presence of *A. baumannii* at a concentration of 1.7 × 10^2^ copies/L. For the effluent samples that showed positive detection of *A. baumannii*, estimated LRVs of 2.7 and 4.2 were calculated for the AeMBR and AnMBR systems, respectively.

Results revealed that *P. aeruginosa* was also present in all influent samples at 5.5 × 10^3^ ± 3.6 × 10^3^ copies/L but was undetected in any AeMBR activated sludge samples (Fig. 2c). Despite not being present in the activated sludge, four of seven AeMBR effluent samples indicated the presence of *P. aeruginosa* at an average *regA* gene concentration of 6.2 × 10^2^ ± 5.6 × 10^2^ copies/L. Two of the 11 AnMBR effluent samples also showed positive detection with an average concentration of 3.3 × 10^2^. The LRV rates for the AeMBR and AnMBR effluent samples indicating *P. aeruginosa* presence were 1.0 and 1.2, respectively.

*K. pneumoniae* was similarly detected in all eight influent samples at an average of 7.4 × 10^5^ ± 4.7 × 10^5^ copies/L (Fig. 2d). This species was further identified in five of eight AeMBR activated sludge samples at a *phoE* gene concentration of 9.7 × 10^4^ ± 3.2 × 10^4^ copies/g. Effluents of both the AeMBR and the AnMBR also showed positive detection for *K. pneumoniae* at 5.9 × 10^2^ ± 6.4 × 10^0^ (3 of 7) and 9.7 × 10^1^ ± 2.0 × 10^1^ (5 of 11), respectively. This resulted in LRVs of 3.1 and 3.9 for *K. pneumoniae* in the AeMBR and AnMBR systems, respectively.

### Quantitative microbial risk assessment

QMRA was performed for *A. baumannii*, *P. aeruginosa*, and *K. pneumoniae* to determine exposure doses imposed by AeMBR and AnMBR effluents on agricultural workers during irrigation activities. Average exposure doses and annual risks were calculated based on 95% confidence intervals and are presented in Table 2. Full calculation results including point risk estimates and upper and lower interval bounds are provided in Appendix S4. Exposure doses from irrigation with AeMBR effluent for *A. baumannii*, *P. aeruginosa*, and *K. pneumoniae* were determined to be 114, 18.8, and 13.5 cells/event, respectively. These point doses resulted in average annual risk estimates of 6.0 × 10^−3^, 1.9 × 10^1^, and 4.2 × 10^−3^, respectively (Table 2). In the case of the AnMBR effluent, event exposure doses were lower than those in AeMBR effluent and calculated to be 0.8, 3.2, and 2.4 cells/event for *A. baumannii*, *P. aeruginosa*, and *K. pneumoniae*, respectively. This resulted in annual risk estimates of 4.3 × 10^−5^, 6.3 × 10^−2^, and 7.3 × 10^−4^, respectively.

**Table 2:** Average exposure dosage and annual risk of pathogenic species at a 95% confidence interval as determined by quantitative microbial risk assessment (QMRA) for irrigation exposure with influent and AeMBR and AnMBR effluents as well as AeMBR activated sludge dermal exposure and ingestion for land application/disposal activities

Exposure dose and annual risk of species	Influent wastewater—exposure	AeMBR effluent—exposure	AnMBR effluent—exposure	AeMBR sludge—exposure	AeMBR sludge—ingestion
*A. baumannii* exp. dose	1.3 × 10^5^	1.1 × 10^2^	8.1 × 10^−1^	3.8 × 10^2^	2.0 × 10^3^
*A. baumannii* annual risk	1.0 × 10^0^	6.0 × 10^−3^	**4.3 × 10** ^**−5**^	5.0 × 10^−3^	2.6 × 10^−2^
*P. aeruginosa* exp. dose	2.9 × 10^2^	1.9 × 10^1^	3.2 × 10^0^	–	–
*P. aeruginosa* annual risk	1.0 × 10^0^	3.2 × 10^−1^	6.3 × 10^−2^	–	–
*K. pneumoniae* exp. dose	3.9 × 10^4^	1.4 × 10^1^	2.4 × 10^0^	6.3 × 10^1^	3.3 × 10^2^
*K. pneumoniae* annu. risk	1.0 × 10^0^	4.2 × 10^−3^	7.3 × 10^−4^	4.9 × 10^−3^	2.6 × 10^−2^

Bold indicates risk level lower than the acceptable microbial risk of 1 x 10^−4^ denotes not applicable because of lack of detection of corresponding microorganism in that sample type

The potential risks associated with the disposal or land application of activated sludge produced by the AeMBR were also evaluated using QMRA for *A. baumannii* and *K. pneumoniae* based on their detection in activated sludge samples (Table 2). Exposure doses were calculated for both dermal exposure and accidental ingestion of sludge by workers or individuals present at disposal/land application sites and used to estimate the annual risks associated with each. Exposure doses from dermal contact during disposal of AeMBR dewatered activated sludge were calculated to be 377 and 63.2 cells/event for *A. baumannii* and *K. pneumoniae*, respectively. These exposure doses resulted in associated annual risk estimates of 5.0 × 10^−3^ and 4.9 × 10^−3^, respectively. Likewise, accidental ingestion doses of dewatered sludge during disposal were calculated for *A. baumannii* and *K. pneumoniae* as 1990 and 334 cells/event, respectively, resulting in respective annual risk estimates of 2.6 × 10^−2^ for both species.

## Discussion

Both the full-scale AeMBR and the lab-scale AnMBR exhibited stable performance throughout the duration of each system’s operation as well as differences in their respective nitrogen conversion rates. Overall microbial community structures of the effluents of each reactor were significantly different from influent wastewater microbial communities as well as from each other (Fig. 3), implying that reactor type and water quality parameters can significantly affect effluent microbial community dynamics. The situational differences in the scale and operational conditions between the two systems could have also significantly affected their associated microbial communities. Nonetheless, a range of pathogen-associated bacterial genera were found in the effluents of both systems at varying removal rates based on average influent wastewater concentrations.

These findings confirm those of previous studies which indicate that although MBRs provide higher microbial removal rates than conventional wastewater treatment systems, effluents still contain detectable levels of potentially harmful bacteria (Francy et al. 2012; Ghayeni et al. 1999; Zhang and Farahbakhsh 2007). The presence of bacteria in MBR effluents is likely due to the fact that absolute pore sizes of membranes are larger than their nominal values, resulting in a lack of total removal based on size exclusion (Arkhangelsky et al. 2012; Hirani et al. 2010). Another factor affecting the passage of bacteria through MF membranes is their potential deformability under pressure filtration (Helling et al. 2017). The transmission of these cells through the membranes used in the MBR process is problematic, especially because of the potential for regrowth in stored wastewater effluents (Giannakis et al. 2014). The utilization of ultrafiltration (UF) or other higher rejection membranes generally result in lower bacterial effluent concentrations, but the majority of MBR systems still employ MF-type membranes due to their lower operational costs (Arévalo et al. 2012).

Existing research on the bacterial removal capacities of MBRs using culture-based methods has indicated that overall LRVs of total coliforms, *E. coli*, and *Enterococcus* are in the ranges of 5.5–6, 4.5–6, and 4.6–6.2, respectively (Marti et al. 2011; Ottoson et al. 2006; van den Akker et al. 2014; Zanetti et al. 2010). The AeMBR examined in this study showed removal rates that were in a similar range with a total bacterial LRV of 4.1 and at least six pathogen-associated genera with LRVs of ≥4.5 (Table 1). Nonetheless, all 13 of the pathogen-associated genera present in the influent were also detected in the full-scale AeMBR effluent. LRVs ranged from as low as 2.7 (*Legionella*) to as high as 5.6 (*Arcobacter*). System operating conditions and water quality parameters can potentially contribute to these differences. For example, the observed increase in nitrate concentration between influent to effluent samples could have favored denitrifying groups such as *Pseudomonas* (Carlson and Ingraham 1983) and contributed to their relatively lower LRVs (<3.5).

Pathogen-associated genera in post-AeMBR effluents are a source of risk that can be easily mitigated by subjecting the effluent to chlorine disinfection (Wisniewski 2007). However, given the potential risks associated with disinfection byproducts, there has been recent interest in evaluating whether MBR effluents can be directly reused for irrigation and other applications (Purnell et al. 2016). As a result, the risks arising from pathogenic bacteria detected by dPCR to workers potentially irrigating with the full-scale AeMBR effluent were evaluated using QMRA. Results of this analysis showed that potential annual risks associated with this activity were above 6.0 × 10^−3^ for *A. baumannii*, *P. aeruginosa*, and *K. pneumoniae*. These values were higher than the average acceptable annual risk of infection of 10^−4^ (Smeets et al. 2009). Similar to what has been previously determined for post-secondary-treated effluent from a conventional wastewater treatment plant (Al-Jassim et al. 2015), the findings in this study suggest that despite passing through an MF membrane, post-AeMBR effluent still requires disinfection prior to use for irrigation activities. An additional source of risk associated with irrigation use is that of ingestion of aerosolized pathogens. Although not quantified in this study’s assessment due to the high variability of factors affecting possibility of ingestion (i.e., irrigation system type, solar irradiation, wind speed, and humidity) (Teltsch and Katzenelson 1978), this source of exposure could be significant for pathogens responsible for respiratory infections such as *K. pneumoniae*.

Of the three specific pathogenic species targeted by dPCR, both *A. baumannii* and *K. pneumoniae* were found to be in relatively high abundance in the AeMBR activated sludge (Fig. 2b, d). Conversely, *P. aeruginosa*, although present in both influent and effluent samples of the AeMBR, was not detected in the activated sludge. Previous studies have also found *K. pneumoniae*, but not *P. aeruginosa*, at high concentrations in activated sludge (Dudley et al. 1980; Ju et al. 2016). These findings reiterate the need for an accurate evaluation of pathogen presence in activated sludge due to the environmental risks associated with sludge disposal and land application regulations (McCall et al. 2015; Wéry et al. 2008). Furthermore, due to the wide range of sludge pretreatment practices employed worldwide (Pérez-Elvira et al. 2006), the risk of pathogen exposure during land application of untreated dewatered sludge remains of major concern. The present study evaluated the annual risk of infection by *A. baumannii* and *K. pneumoniae* present in the full-scale AeMBR activated sludge in a dewatered state during sludge disposal/land application practices using QMRA. Results indicated that average annual risk by both dermal exposure and accidental ingestion was above 4 × 10^−3^ for both pathogenic species detected in the AeMBR sludge (Table 2), implying a significant potential health risk for workers and individuals exposed to dewatered sludge during disposal and land application activities. These results highlight the need for proper treatment of activated sludge prior to disposal (e.g., by anaerobic digestion) or by employing alternative technologies capable of sludge production minimization.

One possible alternative approach is the use of AnMBRs for wastewater treatment due to their inherently low sludge production rates. A similar evaluation of LRVs to that which was conducted for the AeMBR was hence also performed for AnMBR effluent to determine if it would be suitable for direct reuse. A relatively smaller number of pathogen-associated genera were detected in the lab-scale AnMBR effluent compared to those in the wastewater influent (5 of 13). The LRVs of those genera, however, ranged from 1.7 (*Stenotrophomonas*) to 3.9 (*Aeromonas*), implying that the operating conditions and effluent parameters of the anaerobic system enrich for specific bacteria while removing others. Of the five pathogen-associated genera identified in the AnMBR effluent, those with the lowest LRVs (*Acinetobacter*, *Pseudomonas*, and *Stenotrophomonas*) have been previously determined to include high ammonia-assimilating species (Sasaki et al. 2005a; Sasaki et al. 2005b). These groups were likely enriched for by the AnMBR’s limited nitrification capacity (Table S4). Furthermore, given that all of the genera found in the effluent except for *Acinetobacter* are known to be either strictly or facultative anaerobic, the system’s anoxic conditions likely facilitated the survival of these bacteria. *Acinetobacter*, a strictly aerobic bacterium, has been known to exhibit rapid adaptability and survival in anaerobic conditions (Zafiri et al. 1999), which potentially allowed for its persistence through the AnMBR system while other aerobic genera were fully removed.

Despite showing LRVs at the genus and family level in the range of 2.5–2.9 (Table 1), pathogenic species associated with *Acinetobacter* and *Enterobacteriaceae* (as detected by dPCR) showed higher removal in the AnMBR (LRVs of 4.2 and 3.9, respectively). These findings imply that although the effluent of the AnMBR likely enriched for several pathogen-associated genera, the abundances of their respective pathogenic species could be significantly lower. QMRA analysis of the AnMBR effluent for irrigation activities revealed that the annual risk of infection by *A. baumannii* was below the annual acceptable limit (4.3 × 10^−5^). Conversely, *K. pneumoniae* was slightly above that threshold (7.3 × 10^−4^), while the annual risk for infection by *P. aeruginosa* was determined to be more substantial (6.3 × 10^−2^). These results imply that, compared to AeMBR effluent, chlorination may be less crucial for the AnMBR effluent. This is useful for reducing the formation of disinfectant by-products without significantly compromising associated microbial risks. However, for selected microbial groups (e.g., *P. aeruginosa*), additional process optimization measures or better management practices would be necessary to minimize occupational hazards and public health concerns.

One limitation of the present study, and DNA-based monitoring in general, is the potential for overestimation of bacterial abundance due to the presence of nonviable cells and/or extracellular DNA. There are steps, however, that can be taken to minimize the inclusion of DNA from these sources when preparing samples for extraction. For example, the material and pore size of the filters used in this study to retain bacterial cells for extraction have been previously shown to minimize the amount of extracellular DNA included (Liang and Keeley 2013). Although the observed gene abundances of influent wastewater samples in the present study are similar to those observed in a previous study employing qPCR (Shannon et al. 2007), there are other techniques that can be used to further improve the estimation of viable bacteria. For example, the coupling of propidium monoazide with qPCR has been used in various studies to determine the presence of presumably viable cells with intact membranes (Bae and Wuertz 2009; Taskin et al. 2011; van Frankenhuyzen et al. 2011). Nonetheless, when considering the use of molecular techniques for pathogen detection and risk estimation in lieu of culture-based methods, these inconsistencies and potential for overestimation of actual risks should be taken into account.

Another important limitation is that this study cannot be considered a comparison of AeMBRs and AnMBRs for pathogen removal, but is rather a case study of two systems with vastly different operational parameters. Nonetheless, the conclusions drawn from this study regarding the lab-scale AnMBR and its removal capacity are relevant to evaluating it as an alternative municipal wastewater treatment system. The AnMBR was generally effective at removing the pathogenic species targeted by dPCR with only a small number of the total effluent samples showing positive detection and relatively higher LRVs for those samples (Fig. 2). Furthermore, the ability of anaerobic reactors to convert municipal waste to energy instead of sludge indicates that AnMBRs may be advantageous in addressing the microbial-based problems associated with wastewater treatment and sludge disposal.

## Conclusions

Despite the inherent differences in scale and operational conditions between the two MBRs studied, specific removal rates of pathogens by MF-based MBRs can vary significantly between species regardless of the system employed. All of the pathogen-associated genera detected in the influent were also identified in the full-scale AeMBR effluent with a wide range of LRVs. The AnMBR was generally effective at removing the pathogenic species targeted by dPCR with only a small number of effluent samples showing positive detection. Nonetheless, QMRA analysis showed that despite favorable removal rates, direct reuse of the MBR effluents could still pose a substantial risk to humans. Likewise, the activated sludge produced from the AeMBR plant introduces an additional risk arising from land application or disposal practices. These findings emphasize the necessity for a comprehensive understanding of pathogenic removal rates from influent, as well as pathogenic presence in sludge and effluents through molecular-based approaches.

## Electronic supplementary material

ESM 1 (DOCX 4702 kb)
