# Supplementary material for: Molecular-based detection of potentially pathogenic bacteria in membrane bioreactor (MBR) systems treating municipal wastewater: a case study
Source: Environ Sci Pollut Res Int. 2016 Dec 24;24(6):5370–80. doi: 10.1007/s11356-016-8211-y (PMC5352760; doi:10.1007/s11356-016-8211-y)

**Supplementary material:**

**“Molecular-based detection of potentially pathogenic bacteria in membrane bioreactor (MBR) systems treating municipal wastewater: a case study”**

**Moustapha Harb ^1^ and Pei-Ying Hong ^1*^**

^1^ Water Desalination and Reuse Center, Environmental Science and Engineering,

King Abdullah University of Science and Technology (KAUST)

Email: peiying.hong@kaust.edu.sa

**Contents:**

**Appendix S1:** System operating conditions for AeMBR and AnMBR

**Appendix S2:** 16S rRNA gene-based next generation sequencing description

**Appendix S3:** Digital PCR (dPCR) detection of pathogenic species description

**Appendix S4:** Quantitative microbial risk assessment (QMRA) description and calculations

**Table S1:** Sequences of oligonucleotide primers used

**Table S2:** Composition of synthetic wastewater supplement

**Table S3:** Influent and effluent water quality parameters of full-scale AeMBR

**Table S4:** Influent and effluent water quality parameters and biogas production of AnMBR

**Figure S1:** Calculation of detected copy numbers versus expected for known dilutions

**Appendix S1**

**Full-scale AeMBR WWTP operational conditions**

Total wastewater treated per day is 6700 m^3^. The anoxic tank is approximately 350 m^3^ in volume and the activated sludge constitutes a total volume of 1600 m^3^. Mixed liquor suspended solids (MLSS) concentration of the activated sludge is maintained at approximately 16 g/L. The membrane tank system is comprised of 4 equal volume membrane tanks, each of which has a total membrane surface area of 4000 m^2^. The treatment system’s overall HRT and sludge retention time (SRT) are 7 h and 40 d, respectively. Flat-sheet membrane cartridges by Kubota Membrane (Kubota Corporation, Osaka, Japan) are used in this MBR system. The cartridges contain microfiltration membranes with a nominal pore size of 0.4 μm and 0.8 m^2^ of effective surface area per cartridge. Membranes are operated at a flux of approximately 15.5 L/m^2^/h and a trans-membrane pressure of below 20 kPa. The membranes have been in service for 6 years and are cleaned monthly using 5% ClO^-^ solution. Samples were collected approximately once a month over a 10-month period at a minimum of one week after any membrane cleaning event. Influent samples were collected after the primary clarifier, effluent was collected at the membrane permeate discharge point, and activated sludge was collected from the aerobic tank.

**Lab-scale AnMBR operational conditions and sampling**

The lab-scale AnMBR used in this study was filled with cylindrical ceramic packing media (1.5 cm diameter and length). The system was maintained at a temperature of 35 °C (mesophilic conditions). An external cross-flow flat sheet membrane module was used with a polyvinylidene difluoride (PVDF) microfiltration (MF) membrane. The PVDF membrane used was a JX model MF PVDF (GE Osmonics, Minnetonka, MN, USA). Average pore size was 0.3 μm as determined by the manufacturer. The membrane module had an effective membrane surface area of 50 cm^2^ and was operated with a recirculation to permeate ratio of 600:1. Trans-membrane flux was maintained between 6 and 7 L/m^2^/h with an average hydraulic retention time (HRT) of 48 h. The membrane was not cleaned or replaced during operation and trans-membrane pressure (TMP) was below 50 kPa throughout. Biogas was used to scour the membrane surfaces continuously at a biogas to suspended sludge volume ratio of 1:4. Sludge was not wasted except as needed for sampling resulting in a sludge retention time (SRT) of 355 d. MLSS was not quantified for the suspended sludge of the AnMBR, as it was an attached-growth system and the majority of the anaerobic sludge was attached to the ceramic packing media and not in suspension. The AnMBR was fed with the same municipal wastewater reaching the full-scale MBR (130-190 mg/L COD). The wastewater supplemented with synthetic organic media used previously (Harb et al. 2015) in order to maintain robust anaerobic MBR operational conditions. This resulted in a final influent COD of 1700-1850 mg/L COD and an organic loading rate of 0.75-0.85 g/L/d. The NH_3_ concentration was maintained at approximately 304 mg/L (±250 mg/L NH_3_-N) so as to be proportionally representative of the final influent wastewater COD. Feed wastewater was stored at 4 °C and changed every 16 h to maintain microbial stability. The AnMBR was acclimated and run for several months prior to the commencement of sampling.

**Appendix S2**

**16S rRNA gene-based next generation sequencing**

515F (5’- Illumina overhang- GTG YCA GCM GCC GCG GTA A- 3’) and 907R (5’- Illumina overhang- CCC CGY CAA TTC MTT TRA GT- 3’) primers were modified to encode the overhang adaptor sequences, and used to amplify for the 16S rRNA genes. The thermal cycling program included an initial denaturation stage at 95 °C for 3 min, 25 cycles of denaturation (95 °C for 30 s), annealing (55 °C for 30 s) and extension (72 °C for 30 s), and then a final extension stage at 72 °C for 5 min. PCR amplicons were purified by AMPure XP beads (Beckman Coulter, CA). Nextera XT Index (Illumina, San Diego, CA) was incorporated into each of the individual samples during PCR. The indexing thermal cycling program included an initial denaturation stage at 95 °C for 3 min, 8 cycles of denaturation (95 °C for 30 s), annealing (55 °C for 30 s) and extension (72 °C for 30 s), and then a final extension stage at 72 °C for 5 min. The final indexed PCR amplicons were again purified by AMPure XP beads and quantified for the concentrations using an Invitrogen Qubit® 2.0 fluorometer. Controls for all PCR reactions were negative for amplification.

**Appendix S3**

**Determination of bacterial species abundance by digital PCR (dPCR)**

The Clarity^TM^ digital PCR system works with a fluorescently tagged probe as the basis for template quantification. Each PCR reaction mix volume is partitioned into 10000 sub-reactions on a high-density chip using the Clarity^TM^ Auto-Loader and the Clarity^TM^ Sealing Enhancer. Each partitioned reaction is then subjected to thermal cycling, after which the fluorescence from each partition is detected by the Clarity^TM^ Reader. Based on the appropriate dilution of DNA template samples, each partition should contain a maximum of one DNA molecule. Partitions with the target template DNA would be amplified, yielding a positive fluorescence signal that would differentiate against partitions without the target template DNA. By counting the number of positive fluorescence signals, the concentration of the target template DNA can be calculated. To account for the possibility of multiple DNA molecules being present in one partition, values were adjusted based on a Poisson distribution equation previously described (Pinheiro et al. 2011).

All samples and no template controls (NTCs) were run in duplicate. All NTCs were run alongside the samples in each PCR and reading sequence. Each reaction volume of 15 μL contained 7.5 µL of Biotium® EvaGreen® Master Mix (Biotium Inc., Hayward, CA, USA), 0.75 μL of JN Solution (JN Medsys), 0.1 µL of each primer (10 µM), 3 µL of DNA template from each sample and 3.55 µL H_2_O. The thermal cycling protocol in the standard mode comprised of 1 cycle of 96 ^o^C for 5 min, 40 cycles of 96 ^o^C for 50 sec and 59 ^o^C for 1 min 30 sec, and post-PCR hold temperature of 28 ^o^C. Positive reads were determined as a fraction of the total loaded partitions and used to calculate the DNA copy concentration per volume of PCR mix. Relative gene abundances were then normalized per liter of sample based on the original extracted volume of each sample and the volume eluted during DNA extraction. All NTCs of targeted *ompA*, *phoE*, *regA*, and *rpoB* genes were negative with less than two positive reads (<0.80 copies per µL) partition chip.

To determine the sensitivity of the platform, standard dilutions of known concentrations of each gene target were also run using PCR standards created by cloning the target gene into pCR®2.1-TOPO vectors (Invitrogen, Inc., Carlsbad, CA, USA) using protocols described previously (Ansari et al. 2015). Standards were created at dilutions of 10^4^, 10^3^, 10^2^, and 10^1^ for each gene target. Standard dilutions were reproducibly distinguishable against background noise and false positives for all genes at levels as low as 2 copies per µL of stock DNA. Standard curves based on the above serial dilution factors and the calculated known DNA concentrations as measured by a Qubit 2.0 fluorometer (Thermo Fisher Scientific, Carlsbad, CA) were used to compare the expected copies per µL against the detected copies per µL for target genes (R^2^ > 0.99). The detected copies and actual copies spiked in each standard were plotted (Figure S3) and used to determine separation along the x-axis. Therefore, x-intercepts represent the difference between the known DNA concentrations of each gene and the readings obtained by dPCR for each primer. Adjustments based on each gene’s intercept separation values were therefore made by multiplying the readings obtained by dPCR by their respective x-intercepts so as to obtain the actual concentrations of each gene (Figure S3).

**Appendix S4**

**Quantitative microbial risk assessment (QMRA):** Explanation of the QMRA calculations conducted for *Acinetobacter baumannii, Pseudomonas aeruginosa,* and *Klebsiella pneumoniae* are based on typical irrigation practices, sludge production and disposal values for the local MBR wastewater treatment plant, and the USEPA exposure factors handbook (USEPA 2011). For irrigation-based exposure to the MBR effluents, 33% of the total 15310 cm^2^ body surface area was assumed to be exposed. 0.146 mL/cm^2^ aqueous particles were assumed to adhere to the exposed body surface. An irrigation frequency of 4 days per week with 20-minute irrigation per event and 180 mL per m^2^ of land were also assumed. In the case of land application of dewatered activated sludge, a sludge disposal mass of 4200 kg per event, 0.1 mg of adhered sludge per kg disposed, and land application activity duration of 2 h per week were assumed. Dose exposures of hands, face, arms and feet were calculated based on surface area and dermal adherence USEPA values. Ingestion values were based on a hand to eye/lip/nostril contact frequency value of 15.7. An assumed 2.0 x 10^-6^ probability of transmission of bacteria to host was used (Gerba and Choi 2006) for all three pathogenic species assessed.

| **Parameters for irrigation** | **Annotation** | **Assumed value** | **Ref.** |
| --- | --- | --- | --- |
| Area of land occupied by farmer during irrigation (m^2^) | A | 10 |  |
| Water usage (ml/min/m^2^) | B | 180 |  |
| Irrigation frequency (days per week) | C | 4 |  |
| Duration of irrigation (min/event) | D | 20 |  |
| Fraction of total body surface area exposed | E | 0.33 | (USEPA 2011) |
| Total body surface area (cm^2^) | F | 15310 |  |
| Liquid volume adhered onto exposed skin (ml/cm^2^) | G | 0.14635 |  |
| Transmission probability of bacterium to host | H | 2.00 x 10^-6^ | (Gerba and Choi 2006) |

| **Parameters for sludge exposure** | **Annotation** | **Assumed value** | **Ref.** |
| --- | --- | --- | --- |
| Sludge disposal mass (kg/week) | I | 4200 |  |
| Sludge contacted per disposal by worker (mg/kg) | J | 0.1 |  |
| Exposure activity (h/week) | K | 2 |  |
| Skin exposure – Hands (mg/h) | L | 208.9 | (USEPA 2011) |
| Skin exposure – Arms (mg/h) | M | 151.2 |  |
| Skin exposure – Face (mg/h) | N | 36.9 |  |
| Skin exposure – Feet (mg/h) | O | 224.3 |  |
| Hand – eye/lip/nostril contact (times/h) | P | 15.7 |  |
| Transmission probability of bacterium to host | Q | 2.00 x 10^-6^ | (Gerba and Choi 2006) |

| ***A. baumannii* Parameters** | **Annotation** | **Type of wastewater** | | | **Ref.** |
| --- | --- | --- | --- | --- | --- |
|  |  | **AeMBR Effluent** | **AnMBR Effluent** | **Activated Sludge** |  |
| Average cell numbers of *A.* *baumannii* per mL (or mg) | R | 4.99 | 0.167 | 361 |  |
| Occurrence rate fraction of *A.* *baumannii* in all samples | S | 0.43 | 0.09 | 1 |  |
| Irrigation exposure dose (cells/event)**, X**  = A * B * C * D * E* F * G * H * R * S | | 114 | 0.808 | - |  |
| Sludge exposure dose (cells/event)**, X**  = I * J * K * (L + M + N + O) * Q * R * S | | - | - | 376 |  |
| Sludge ingestion dose (cells/event)**, X**  = I * J * K * L * P * Q * R * S | | - | - | 1989 |  |
| LD_50_ (CFU) | | 2.51 x 10^6^ | | | (López-Rojas et al. 2011) |
| k (exponential model) | | 2.76 x 10^-7^ | | |  |
| Point estimate of risk = 1 – exp (-k *exp. dose) | | 3.14 x 10^-5^ | 2.23 x 10^-7^ | 1.04 x 10^-4^ |  |
| Point estimate of risk = 1 – exp (-k *inj. dose) | |  | | 5.49 x 10^-4^ |  |
| Annual risk exposure =  1- (1-point estimate)^(C * number of weeks * number of months) | | 6.02 x 10^-3^ | 4.29 x 10^-5^ | 4.98 x 10^-3^ |  |
|  |  |  |  | 2.60 x 10^-2^ |  |

| ***P. aeruginosa* Parameters** | **Annotation** | **Type of wastewater** | | | **Ref.** |
| --- | --- | --- | --- | --- | --- |
|  |  | **AeMBR Effluent** | **AnMBR Effluent** | **Activated Sludge** |  |
| Average cell numbers of *P.* *aeruginosa* per mL (or mg) | R | 0.617 | 0.334 | - |  |
| Occurrence rate fraction of *P.* *aeruginosa* in all samples | S | 0.57 | 0.19 | - |  |
| Exposure dose (cells/event)**, X**  = A * B * D * E* F * G * H * R * S | | 18.8 | 3.23 | - |  |
| LD_50_ (CFU) | | 6.61 x 10^3^ | | | (Hazlett et al. 1978) |
| k (exponential model) | | 1.05 x 10^-4^ | | |  |
| Point estimate of risk =  1 – exp (-k * exposed dose) | | 1.97 x 10^-3^ | 3.39 x 10^-4^ | - |  |
| Annual risk =  1- (1-point estimate)^(C * number of weeks * number of months) | | 3.15 x 10^-1^ | 6.30 x 10^-2^ | - |  |

| ***K. pneumoniae* Parameters** | **Annotation** | **Type of wastewater** | | | **Ref.** |
| --- | --- | --- | --- | --- | --- |
|  |  | **AeMBR Effluent** | **AnMBR Effluent** | **Activated Sludge** |  |
| Average cell numbers of *K.* *pneumoniae* per mL (or g) | R | 0.591 | 0.0970 | 96.9 |  |
| Occurrence rate fraction of *K.* *pneumoniae* in all samples | S | 0.43 | 0.45 | 0.63 |  |
| Irrigation exposure dose (cells/event)**, X**  = A * B * C * D * E* F * G * H * R * S | | 13.5 | 2.35 | - |  |
| Sludge exposure dose (cells/event)**, X**  = I * J * K * (L + M + N + O) * Q * R * S | | - | - | 63.2 |  |
| Sludge ingestion dose (cells/event)**, X**  = I * J * K * L * P * Q * R * S | | - | - | 334 |  |
| LD_50_ (CFU) | | 4.29 x 10^5^ | | | (Domenico et al. 1982) |
| k (exponential model) | | 1.62 x 10^-6^ | | |  |
| Point estimate of risk = 1 – exp (-k *exp. dose) | | 2.18 x 10^-5^ | 3.79 x 10^-6^ | 1.02 x 10^-4^ |  |
| Point estimate of risk = 1 – exp (-k *inj. dose) | |  | | 5.39 x 10^-4^ |  |
| Annual risk exposure =  1- (1-point estimate)^(C * number of weeks * number of months) | | 4.17 x 10^-3^ | 7.28 x 10^-4^ | 4.89 x 10^-3^ |  |
|  |  |  |  | 2.55 x 10^-2^ |  |

| Irrigation risk bounds  Median, upper and lower | AeMBR Effluent - exposure | | | AnMBR Effluent - exposure | | |
| --- | --- | --- | --- | --- | --- | --- |
|  | Med. | Upper | Lower | Med. | Upper | Lower |
| *A.* *baumannii* exp. dose | 1.14 x 10^2^ | 1.71 x 10^2^ | 7.59 x 10^1^ | 8.08 x 10^-1^ | 1.21 x 10^0^ | 5.40 x 10^-1^ |
| *A.* *baumannii* point risk | 3.14 x 10^-5^ | 9.43 x 10^-5^ | 1.05 x 10^-5^ | 2.23 x 10^-7^ | 6.70 x 10^-7^ | 7.44 x 10^-8^ |
| *A.* *baumannii* annual risk | 6.02 x 10^-3^ | 1.79 x 10^-2^ | 2.01 x 10^-3^ | 4.29 x 10^-5^ | 1.29 x 10^-4^ | 1.43 x 10^-5^ |
| *P.* *aeruginosa* exp. dose | 1.88 x 10^1^ | 2.82 x 10^1^ | 1.25 x 10^1^ | 3.23 x 10^0^ | 4.85 x 10^0^ | 2.16 x 10^0^ |
| *P.* *aeruginosa* point risk | 1.97 x 10^-3^ | 5.89 x 10^-3^ | 6.56 x 10^-4^ | 3.39 x 10^-4^ | 1.02 x 10^-3^ | 1.13 x 10^-4^ |
| *P.* *aeruginosa* annual risk | 3.15 x 10^-1^ | 6.78 x 10^-1^ | 1.18 x 10^-1^ | 6.30 x 10^-2^ | 1.77 x 10^-1^ | 2.15 x 10^-2^ |
| *K. pneumoniae* exp. dose | 1.35 x 10^1^ | 2.02 x 10^1^ | 8.99 x 10^0^ | 2.35 x 10^0^ | 3.52 x 10^0^ | 1.56 x 10^0^ |
| *K. pneumoniae* point risk | 2.18 x 10^-5^ | 6.54 x 10^-5^ | 7.26 x 10^-6^ | 3.79 x 10^-6^ | 1.14 x 10^-5^ | 1.26 x 10^-6^ |
| *K. pneumoniae* annu. risk | 4.17 x 10^-3^ | 1.25 x 10^-2^ | 1.39 x 10^-3^ | 7.28 x 10^-4^ | 2.18 x 10^-3^ | 2.43 x 10^-4^ |

| Sludge risk bounds  Median, upper and lower | AeMBR sludge risk - exposure | | | AeMBR sludge risk - ingestion | | |
| --- | --- | --- | --- | --- | --- | --- |
|  | Med. | Upper | Lower | Med. | Upper | Lower |
| *A.* *baumannii* exp. dose | 3.77 x 10^2^ | 5.65 x 10^2^ | 2.51 x 10^2^ | 1.99 x 10^3^ | 2.98 x 10^3^ | 1.33 x 10^3^ |
| *A.* *baumannii* point risk | 1.04 x 10^-4^ | 3.12 x 10^-4^ | 3.47 x 10^-5^ | 5.49 x 10^-4^ | 1.64 x 10^-3^ | 1.83 x 10^-4^ |
| *A.* *baumannii* annual risk | 4.98 x 10^-3^ | 1.48 x 10^-2^ | 1.66 x 10^-3^ | 2.60 x 10^-2^ | 7.60 x 10^-2^ | 8.75 x 10^-3^ |
| *K. pneumoniae* exp. dose | 6.32 x 10^1^ | 9.48 x 10^1^ | 4.21 x 10^1^ | 3.34 x 10^2^ | 5.01 x 10^2^ | 2.23 x 10^2^ |
| *K. pneumoniae* point risk | 1.02 x 10^-4^ | 3.06 x 10^-4^ | 3.40 x 10^-5^ | 5.39 x 10^-4^ | 1.61 x 10^-3^ | 1.79 x 10^-4^ |
| *K. pneumoniae* annu. risk | 4.89 x 10^-3^ | 1.46 x 10^-2^ | 1.63 x 10^-3^ | 2.55 x 10^-2^ | 7.47 x 10^-2^ | 8.59 x 10^-3^ |

**References**

Ansari, M.I., Harb, M., Jones, B. and Hong, P.-Y. (2015) Molecular-based approaches to characterize coastal microbial community and their potential relation to the trophic state of Red Sea. Scientific reports 5.

Dahllöf, I., Baillie, H. and Kjelleberg, S. (2000) rpoB-based microbial community analysis avoids limitations inherent in 16S rRNA gene intraspecies heterogeneity. Applied and environmental microbiology 66(8), 3376-3380.

Domenico, P., Johanson, W. and Straus, D. (1982) Lobar pneumonia in rats produced by clinical isolates of Klebsiella pneumoniae. Infection and immunity 37(1), 327-335.

Gerba, C.P. and Choi, C.Y. (2006) Viruses in Foods. Goyal, S.M. (ed), p. 345, Springer.

Harb, M., Xiong, Y., Guest, J., Amy, G. and Hong, P.-Y. (2015) Differences in microbial communities and performance between suspended and attached growth anaerobic membrane bioreactors treating synthetic municipal wastewater. Environmental Science: Water Research & Technology 1(6), 800-813.

Hazlett, L.D., Rosen, D.D. and Berk, R.S. (1978) Age-Related Susceptibility to Pseudomonas-Aeruginosa Ocular Infections in Mice. Infection and Immunity 20(1), 25-29.

Lee, D.-Y., Shannon, K. and Beaudette, L.A. (2006) Detection of bacterial pathogens in municipal wastewater using an oligonucleotide microarray and real-time quantitative PCR. Journal of microbiological methods 65(3), 453-467.

López-Rojas, R., Domínguez-Herrera, J., McConnell, M.J., Docobo-Peréz, F., Smani, Y., Fernández-Reyes, M., Rivas, L. and Pachón, J. (2011) Impaired virulence and in vivo fitness of colistin-resistant Acinetobacter baumannii. Journal of Infectious Diseases 203(4), 545-548.

McConnell, M.J., Pérez-Ordóñez, A., Pérez-Romero, P., Valencia, R., Lepe, J.A., Vázquez-Barba, I. and Pachón, J. (2012) Quantitative real-time PCR for detection of Acinetobacter baumannii colonization in the hospital environment. Journal of clinical microbiology 50(4), 1412-1414.

Pinheiro, L.B., Coleman, V.A., Hindson, C.M., Herrmann, J., Hindson, B.J., Bhat, S. and Emslie, K.R. (2011) Evaluation of a droplet digital polymerase chain reaction format for DNA copy number quantification. Analytical chemistry 84(2), 1003-1011.

USEPA (2011) Exposure Factors Handbook 2011 USEPA (ed), Washington, DC.

**Table S1.** Sequences of oligonucleotide primers used in this study.

| Primer | | Target Gene | Sequence (5’ – 3’) | Targeted Bacterial Species | Targeted Gene Function | Reference |
| --- | --- | --- | --- | --- | --- | --- |
|  | OmpA-F | *ompA* | TCTTGGTGGTCACTTGAAGC | *Acinetobacter baumannii* | Outer membrane protein A | (McConnell et al. 2012) |
|  | OmpA-R |  | ACTCTTGTGGTTGTGGAGCA |  |  |  |
|  | Kpneu-F | *phoE* | CCTGGATCTGACCCTGCAGTA | *Klebsiella pneumoniae* | Outer membrane phosphate porin | (Lee et al. 2006) |
|  | Kpneu-R |  | GAAACAGAACGGCGACGG |  |  |  |
|  | Paer-F | *regA* | TGCTGGTGGCACAGGACAT | *Pseudomonas aeruginosa* | Toxin A synthesis | (Lee et al. 2006) |
|  | Paer-R |  | TTGTTGGTGCAGTTCCTCATTG |  |  |  |
|  | rpoB-F | *rpoB* | AACATCGGTTTGATCAAC | All Bacteria | RNA polymerase beta subunit | (Dahllöf et al. 2000) |
|  | rpoB-R |  | CGTTGCATGTTGGTACCCAT |  |  |  |

**Table S2.** Composition of synthetic wastewater added to influent in mg/L (1600 mg/L COD, pH 7).

| Carbon source | | Nutrients and Iron | | Trace Metals | |
| --- | --- | --- | --- | --- | --- |
| Starch | 476.2 | Urea | 358.0 | Cr(NO_3_)_3_^.^9H_2_O | 3.0 |
| Milk Power | 453.4 | NH_4_Cl | 49.76 | CuCl_2_^.^2H_2_O | 2.10 |
| Yeast | 203.8 | MgHPO_4_^.^3H_2_O | 113.24 | MnSO_4_^.^H_2_O | 0.42 |
| Peptone | 67.94 | KH_2_PO_4_ | 91.32 | NiSO_4_^.^6H_2_O | 1.33 |
| Na-acetate^.^3H_2_O | 513.8 | FeSO_4_^.^7H_2_O | 22.64 | PbCl_2_ | 0.40 |
|  |  |  |  | ZnCl_2_ | 0.82 |

**Table S3.** Influent and effluent water quality parameters of full-scale AeMBR samples.

|  | Inf. COD  (mg/L) | Eff. COD  (mg/L) | Inf. NH_4_-N  (mg/L) | Eff. NH_4_-N  (mg/L) | Inf.  NO_2_-N  (mg/L) | Eff.  NO_2_-N  (mg/L) | Inf.  NO_3_-N  (mg/L) | Eff.  NO_3_-N  (mg/L) |
| --- | --- | --- | --- | --- | --- | --- | --- | --- |
| 8-Aug-15 | 172 | <10 | 9 | 0 | 4.7 | 0 | 1.55 | 17.4 |
| 22-Oct-15 | 161 | <10 | 12.8 | 0.1 | 0.09 | 0 | 0 | 16.7 |
| 23-Nov-15 | 129 | <10 | 9.4 | 0 | 2.5 | 0 | 0.71 | 10.9 |
| 27-Dec-15 | 145 | <10 | 15.6 | 0 | 0.35 | 0 | 0 | 14.4 |
| 26-Jan-16 | 202 | <10 | 13 | 0 | 0 | 0 | 0 | 16.8 |
| Average | 162±27.7 | <10 | 12.0±2.8 | 0.02±0.04 | 1.5±2.1 | 0 | 0.45±0.69 | 15.2±2.7 |

|  | Inf. COD  (mg/L) | Eff. COD  (mg/L) | Inf. NH_4_-N  (mg/L) | Eff. NH_4_-N  (mg/L) | CH_4_ in biogas  (%) | CH_4_ total (mL CH_4_ / g COD) |
| --- | --- | --- | --- | --- | --- | --- |
| 14-Jul-15 | 1851 | 28 | 255 | 240 | 72.5 | 253 |
| 1-Oct-15 | 1798 | 89 | 248 | 238 | 75 | 248 |
| 5-Nov-15 | 1739 | 46 | 254 | 247 | 78 | 231 |
| 10-Dec-15 | 1768 | 77 | 250 | 241 | 71.4 | 232 |
| Average | 1789±48 | 60±28 | 252±3.3 | 242±3.9 | 74.2±2.9 | 241±12 |

**Table S4.** Influent and effluent water quality parameters and biogas production of AnMBR.

**Figure S1.** Calculation of detected copy numbers per µL versus expected for known dilutions of *ompA*, *regA*, and *phoE* genes.
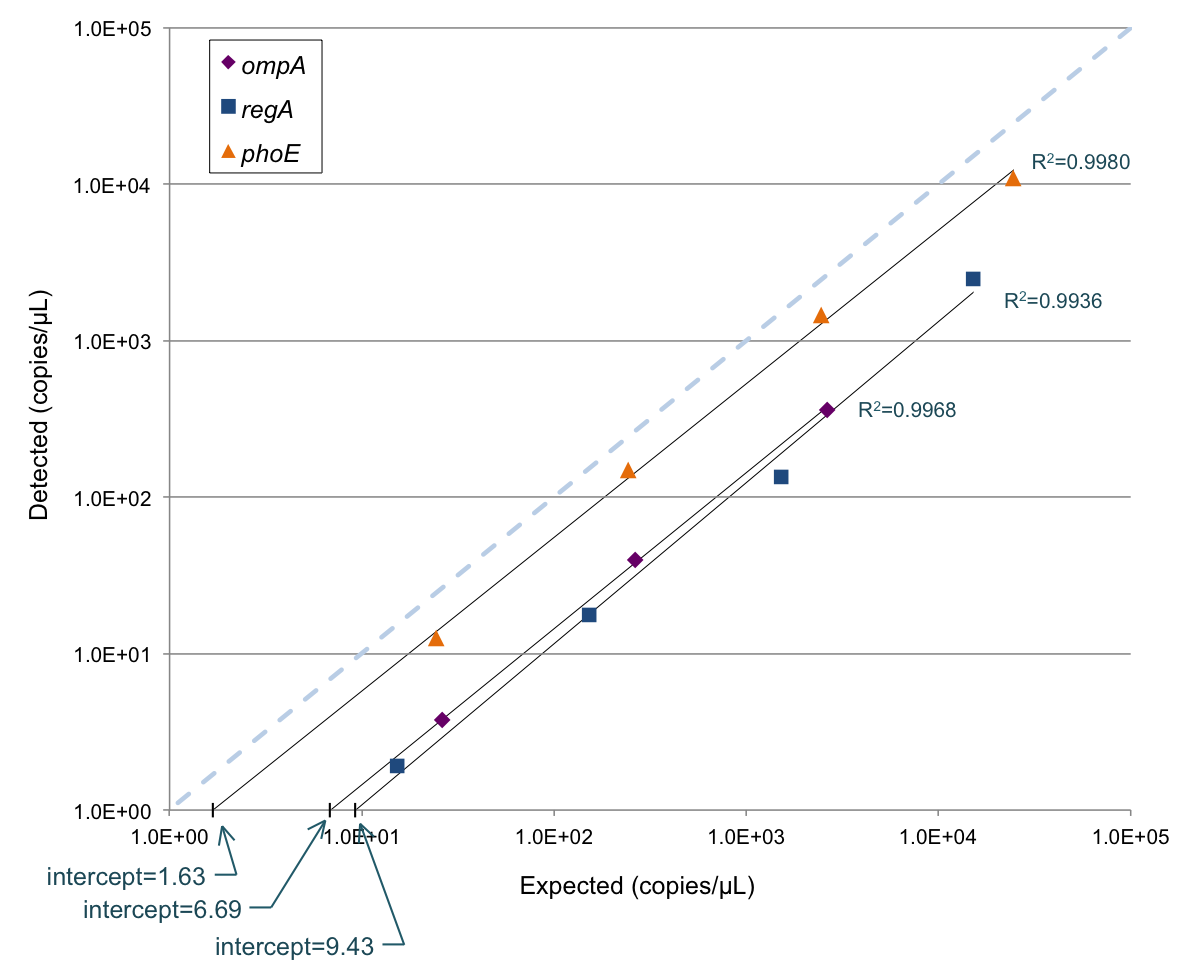

Supplement: Supplementary file 1 — (DOCX 4702 kb) [file 11356_2016_8211_MOESM1_ESM.docx]
